# Comprehensive Performance Verification of the Mindray MN2880 System in Detecting Respiratory Virus Nucleic Acids

**DOI:** 10.1002/jcla.70390

**Published:** 2026-10-25

**Authors:** Jinliang Chen, Lianying Zeng, Hao Huang, Peisong Chen

**Affiliations:** ^1^ Department of Medical Laboratory Chaozhou People's Hospital Affiliated to Shantou University Medical College Chaozhou Guangdong China; ^2^ Department of Laboratory Medicine People's Hospital of Boluo County Huizhou Guangdong China; ^3^ Department of Laboratory Medicine The First Affiliated Hospital, Sun Yat‐Sen University Guangzhou Guangdong China

**Keywords:** concordance rate, detection limit, nucleic acid testing, performance verification, respiratory viruses

## Abstract

**Objective:**

To verify the clinical performance of the Mindray MN2880 system for simultaneous detection of SARS‐CoV‐2, influenza A virus (IAV), influenza B virus (IBV), and respiratory syncytial virus (RSV) nucleic acids.

**Methods:**

According to the CNAS‐GL039:2019 and CNAS‐GL037:2019 requirements for molecular diagnostic verification, we comprehensively evaluated the Mindray MN2880 multiplex respiratory virus assay across seven critical performance parameters: clinical concordance, limit of detection (LoD), repeatability, precision, cross‐reactivity/interference resistance, daily QC, and contamination control.

**Results:**

The Mindray MN2880 system demonstrated high diagnostic concordance with the reference method, with total percent agreements of 99.51% (95% CI: 99.00%–99.76%) for SARS‐CoV‐2, 99.93% (95% CI: 99.60%–99.99%) for IAV, and 100% for both IBV and RSV, and achieved clinically verified limits of detection (LoD: SARS‐CoV‐2 at 100 copies/mL, IAV/IBV/RSV at 200 copies/mL). Analytical precision was confirmed through 100% detection across negative/low/medium/high concentration panels with ≤ 3% CV for repeatability (Ct values) and ≤ 5% CV for inter‐run precision. The assay exhibited 100% specificity against 6 clinically relevant pathogens, including Human Rhinovirus, parainfluenza virus, adenovirus, *Mycoplasma pneumoniae* , *Haemophilus influenzae* , and *Staphylococcus aureus* , with no observed cross‐reactivity. Moreover, the MN2880 system demonstrated robust interference resistance (100% detection of weakly‐positive samples spiked with seven interferents), stable daily QC performance (100% valid controls across 115 consecutive runs), and 100% contamination prevention.

**Conclusion:**

The MN2880 system satisfies key analytical benchmarks required for diagnostic use in respiratory virus multiplex testing, with performance verified against CNAS‐GL039:2019 and CNAS‐GL037:2019 standards.

## Graphical abstract

This study verified the analytical performance of the fully automated Mindray MN2880 system for the simultaneous detection of SARS‐CoV‐2, influenza A/B viruses, and RSV in 2852 oropharyngeal swab specimens, following CNAS‐GL039:2019, CNAS‐GL037:2019, and ISO 15189:2022 guidelines. The system demonstrated high concordance with reference real‐time PCR (total percent agreement 99.51%–100%), a limit of detection of 100–200 copies/mL, repeatability and precision CVs within 3% and 5%, no cross‐reactivity or interference, and stable daily quality control across 115 consecutive runs. With a closed, sample‐to‐result workflow of approximately 40 min, the MN2880 system is qualified for rapid point‐of‐care molecular testing of respiratory viruses in clinical and primary‐care settings.

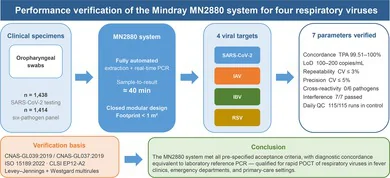

## 1 Introduction

Acute respiratory infections (ARI) represent a significant public health concern globally, contributing to high morbidity and mortality rates, particularly among children and the elderly. These infections are predominantly caused by viruses, with the SARS‐CoV‐2 virus, influenza A viruses (IAV), influenza B viruses (IBV), and respiratory syncytial virus (RSV) currently serving as the key pathogens prioritized in clinical practice and public health surveillance [1, 2]. Surveillance data from Central China revealed a 36.9% viral detection rate in ARI, where influenza virus predominated (13.8%). Pediatric cases showed significantly higher influenza virus positivity (23.9%). The primary circulating viruses were influenza virus, adenovirus, and RSV [3]. International surveillance data demonstrate substantial viral burdens in respiratory infections, evidenced by a 57.4% (914/1592) detection rate in Turkey (2016–2019) dominated by HRV and influenza A [4], and concurrent circulation of RSV (23.7%), HRV (22.3%), and SARS‐CoV‐2 (14.1%) in Northern Italy (2019–2020 winter) [5]. High codetection frequency (19.8%) underscores diagnostic complexity and potential severity escalation [4, 5], mandating accelerated pathogen identification to direct clinical management, infection containment protocols, and public health resource prioritization.

Nucleic acid testing (NAT), particularly nucleic acid amplification techniques (NAAT), has become the cornerstone and gold standard for etiological diagnosis of respiratory viruses due to its exceptional sensitivity and specificity [6]. Although conventional methods, such as real‐time reverse transcription PCR (RT‐qPCR), demonstrate high accuracy, they typically involve complex workflows, prolonged processing times, and require specialized laboratory facilities and trained personnel. These limitations hinder their ability to meet urgent demands for rapid diagnosis in clinical settings, especially emergency departments, outpatient clinics, and primary care facilities [7]. In recent years, molecular diagnostics have advanced toward automated, integrated, and rapid point‐of‐care testing (POCT) platforms. Numerous integrated molecular POCT platforms have been extensively validated for rapid multiplex detection of SARS‐CoV‐2, influenza virus, and RSV. Their performance demonstrates high concordance with laboratory gold standard methods, delivering reliable results within 60 min or less. This capability significantly facilitates rapid differential diagnosis and clinical decision‐making [8, 9, 10, 11, 12, 13, 14, 15]. To ensure reliability, comparability, and accuracy of these rapid automated testing systems in clinical applications, comprehensive and rigorous performance validation and ongoing evaluation must be conducted in compliance with relevant guidelines [16]. Core performance validation parameters encompass analytical sensitivity (limit of detection), analytical specificity (including cross‐reactivity and interfering substances), precision, trueness (or accuracy), and reportable range [15]. For instance, evaluation of the STANDARD M10 Flu/RSV/SARS‐CoV‐2 Fast assay demonstrated detection sensitivities of 98.2% for influenza A, 100% for influenza B, 95.1% for RSV, and 100% for SARS‐CoV‐2, alongside high specificity [15]. Such performance verification is critical for laboratories to select appropriate platforms and ensure testing quality.

In this study, the Mindray MN2880 system is designed specifically for POCT requirements. The Mindray MN2880 is a fully integrated automated platform combining magnetic bead‐based nucleic acid extraction with real‐time fluorescent PCR amplification and detection. This system consolidates multiple procedural steps, including sample lysis, nucleic acid purification, reaction mixture assembly, and amplification analysis, into a unified workflow, delivering end‐to‐end automation from primary tube loading to final result reporting. It performs fully automated nucleic acid purification and real‐time PCR analysis for respiratory viruses, delivering results in as little as 40 min, thereby enabling rapid nucleic acid testing turnaround. This study conducted performance verification of the MN2880 system for detecting four respiratory viruses in strict accordance with CNAS‐GL039:2019 [17], CNAS‐GL037:2019 [18], and the “Technical Review Guidelines for Registration of Multiplex Nucleic Acid Assays for Respiratory Viruses” [19]. Validation parameters included concordance rate, limit of detection (LoD), repeatability, precision, cross‐reactivity/interference resistance, daily quality control, and contamination prevention measures.

## 2 Materials and Methods

### 2.1 Specimen Collection

Oropharyngeal swab specimens were collected at the Department of Clinical Laboratory Medicine, the First Affiliated Hospital of Sun Yat‐sen University from May 2025 to November 2025, comprising 1438 samples for SARS‐CoV‐2 nucleic acid detection and 1414 samples for six respiratory pathogen nucleic acid testing (IAV, IBV, RSV, adenovirus, human rhinovirus, and *Mycoplasma pneumoniae* ). All specimens were residual samples from routine clinical testing; they were stored at 2°C–8°C and tested within 48 h of collection, and residual aliquots were frozen at −70°C (no more than one freeze–thaw cycle) for discordant‐result retesting. The study was approved by the Ethics Committee of the First Affiliated Hospital of Sun Yat‐sen University (Ethics Approval: No. 2024‐554).

### 2.2 Reagents and Instruments

The performance validation utilized the Mindray MN2880 Automated Nucleic Acid Extraction/Real‐time PCR System with its multiplex respiratory PCR assay kit (SARS‐CoV‐2, IAV, IBV, and RSV) as the test platform. SARS‐CoV‐2 detection reagents were sourced from DAAN Gene Co. Ltd. (Guangzhou, China), while the six‐pathogen respiratory panel reagents (influenza A/B, RSV, adenovirus, human rhinovirus, *Mycoplasma pneumoniae* ) originated from Sansure Biotech Inc. (Changsha, China). Nucleic acid extraction employed DAAN's Smart32 automated extractor (DAAN Gene, Guangzhou, China), with the ABI 7500 Real‐Time PCR System (Thermo Fisher Scientific Inc., Waltham, MA, USA) serving as the reference method. Both negative and positive quality control materials for SARS‐CoV‐2, IAV, IBV, and RSV were manufactured by Shenzhen Mindray Bio‐Medical Electronics Co. Ltd., with concentrations validated to comply with their declared limits of detection (LoD).

### 2.3 Executive Standard

Pursuant to the Guidance on Performance Verification for Molecular Diagnostic Testing Procedures (CNAS‐GL039:2019) and Guidance for the Performance Verification of Quantitative Clinical Chemistry Examination Procedures (CNAS‐GL037:2019), the core analytical performance of Mindray's fully automated molecular testing system MN2880 for detecting four respiratory viruses was verified across seven key parameters, including concordance, LoD, repeatability, precision, cross‐reactivity/interference resistance, daily quality control, and contamination prevention.

### 2.4 Concordance Verification

A total of 1438 oropharyngeal swab specimens (previously tested for SARS‐CoV‐2) and 1414 specimens (pretested for six respiratory pathogens: influenza A/B, RSV, adenovirus, rhinovirus, and *M. pneumoniae* ) underwent parallel testing using the Mindray MN2880 multiplex respiratory assay (targeting SARS‐CoV‐2/IAV/IBV/RSV) versus gold‐standard real‐time qPCR; concordant results were accepted when both methods agreed, while discordant specimens underwent definitive confirmation via Sanger sequencing, with validation success defined as ≥ 95% point estimates for positive/negative/overall percent agreement and > 90% lower confidence limits for all agreement metrics at the 95% confidence level. Two‐sided 95% confidence intervals (CI) for all agreement metrics were calculated using the Wilson score method [20]. Discordant specimens were retested in duplicate on both platforms and adjudicated by Sanger sequencing of the target amplicon, and the adjudicated results were used for final classification.

### 2.5 LoD

Validation was performed using four target nucleic acid controls serially diluted to their declared LoD concentrations (SARS‐CoV‐2: 100 copies/mL; IAV, IBV, and RSV: 200 copies/mL). For each target, one reagent lot and one instrument system were tested across five separate days, with quadruplicate runs per day on the MN2880 platform, generating a minimum of 20 replicate determinations. Dilutions were prepared in transport media or commutable negative matrix confirmed negative for all analytes. Verification was considered successful if a positive detection rate of ≥ 95% was achieved (e.g., ≥ 18 out of 20 replicates detecting target nucleic acid).

### 2.6 Repeatability

Verification samples for each pathogen (IAV, IBV, SARS‐CoV‐2, and RSV) were prepared at four concentrations, including negative, low, medium, and high. Concentration thresholds were defined by Ct values on the MN2880 platform: high (Ct ≤ 27), medium (27 < Ct ≤ 33), and low (33 < Ct ≤ 37). These thresholds were chosen because the reportable upper Ct limit of the assay is 37, approximating the declared LoD, whereas Ct ≤ 27 corresponds to moderately high viral loads, so that verification covered the clinically relevant measurement range [20]. For each positive concentration level of each pathogen, three independent samples were each tested in 10 replicates within a single run, and the negative sample was also tested in 10 replicates, using a single reagent lot. Mean Ct values and standard deviations were calculated for all replicates. Verification passed if: negative samples demonstrated a 100% negative detection rate; positive samples achieved a 100% positive detection rate; the coefficient of variation (CV%) for Ct values was ≤ 3%.

### 2.7 Precision

High‐concentration clinical samples/reference materials and negative matrix/reference materials were used to prepare negative, low‐, medium‐, and high‐concentration positive samples with the following Ct value ranges: high (Ct ≤ 27), medium (27 < Ct ≤ 33), and low (33 < Ct ≤ 37) (as defined in the Repeatability section). For each target, ≥ 8.5 mL of sample was prepared, aliquoted equally into five portions (≥ 1.7 mL each), and stored at ≤ −20°C with ≤ 1 freeze–thaw cycle. One analytical run was performed daily for five consecutive days, testing all four concentration levels per run with quintuplicate measurements per level. This generated ≥ 25 measurements per concentration level. Verification passed if: negative samples demonstrated a 100% negative detection rate; positive samples achieved 100% positive detection rate; CV% for Ct values was ≤ 5%.

### 2.8 Cross‐Reaction

Cross‐reactivity was validated using genetically homologous pathogens that cause clinically similar symptoms, including Human rhinovirus, Parainfluenza virus, Adenovirus, *Mycoplasma pneumoniae* , *Haemophilus influenzae* , and *Staphylococcus aureus* , at medically relevant concentrations (1.0 × 10^7^ copies/mL for all pathogens). Each cross‐reactant was spiked into pre‐characterized negative matrix and tested in triplicate on the Mindray MN2880. Validation passed provided that all negative controls remained undetected (100% specificity). The panel was selected to cover pathogens genetically related to the targets or associated with overlapping clinical presentations, as recommended by the referenced registration guidance. In addition, in silico analysis of the primer and probe sequences against other common respiratory pathogens (human coronaviruses 229E, NL63, HKU1, and OC43; human metapneumovirus; *Bordetella pertussis* ; and *Chlamydia pneumoniae* ) revealed no significant homology that would predict cross‐amplification, supporting the empirically observed specificity.

### 2.9 Interference Resistance

Interference testing was performed using common molecular diagnostic interferents, including human blood (10% v/v), mucin (20 μg/mL), oseltamivir (100 μg/mL), peramivir (100 μg/mL), ribavirin (100 μg/mL), ceftriaxone (100 μg/mL), and levofloxacin (100 μg/mL), spiked into low‐positive samples (≥ 7 samples per target with Ct values 33 < Ct ≤ 37) at final concentrations matching package insert claims. Control samples received equivalent solvent volumes. All samples underwent ≥ 3 replicate tests on the MN2880 system. Validation passed if low‐positive samples maintained low‐positive results. In addition, Ct values of all valid paired results were recorded, and the Ct shift (ΔCt, defined as the Ct of the interferent‐spiked sample minus that of the paired control) was analyzed using a paired *t*‐test to quantify any systematic effect.

### 2.10 Daily Quality Control

Negative and positive controls were included in every daily testing batch. The negative control consisted of a matrix solution, free of all target sequences detected by the assay but sharing identical or comparable physicochemical properties with clinical samples, while the positive control contained synthetic target sequences in a medium‐concentration matrix (Ct range: 27 < Ct ≤ 33). Both controls underwent the full sample processing and detection workflow. Validation passed only if the negative control yielded undetectable results and the positive control generated positive results. Positive‐control Ct values from all consecutive runs were further monitored using Levey‐Jennings charts with the mean and ±1SD, ±2SD, and ±3SD control lines, and Westgard multirules were applied for run acceptance, with 1_2s as a warning rule and 1_3s, 2_2s, and R_4s as rejection rules [21, 22].

### 2.11 Contamination Prevention

Four petri dishes containing negative matrix solution were placed at distinct locations within the instrument and exposed to ambient air to collect settling particulates. Matrix solutions from all dishes were sampled at six predetermined time points (Day 1, Day 4, Day 9, Day 14, Day 21, and Day 30) and tested in triplicate. Validation passed only if all samples generated negative results.

## 3 Results

### 3.1 Concordance Verification Data

Validation testing using the Mindray MN2880 system demonstrated concordance rates of 99.53% for SARS‐CoV‐2 negative samples, 99.46% for positive samples, and an overall rate of 99.51%. For IAV, negative concordance reached 99.92% with 100.00% positive concordance and 99.93% overall. Both IBV and RSV exhibited 100.00% concordance across negative, positive, and overall metrics (Table 1), confirming successful verification of all concordance parameters. The 95% CIs (Wilson score) for negative/positive/total percent agreement were: SARS‐CoV‐2, 99.53% (98.91%–99.80%), 99.46% (98.05%–99.85%), and 99.51% (99.00%–99.76%); IAV, 99.92% (99.57%–99.99%), 100% (96.23%–100%), and 99.93% (99.60%–99.99%); IBV, 100% (99.73%–100%), 100% (60.97%–100%), and 100% (99.73%–100%); RSV, 100% (99.71%–100%), 100% (95.19%–100%), and 100% (99.73%–100%). The lower 95% confidence limit of the IBV positive percent agreement (60.97%) did not meet the prespecified > 90% criterion, reflecting the small number of IBV‐positive specimens (*n* = 6). Discordant results were adjudicated by Sanger sequencing, which did not change the overall concordance conclusions.

**TABLE 1:** Concordance validation data between MN2880 and real‐time fluorescence quantitative PCR or sequencing.

MN2880	DAAN/Sansure/Sequencing		Total	PPA (%)	NPA (%)	TPA (%)	Kappa
	P	N					
IAV							
P	98	1	1414	100.00 (96.23–100.00)	99.92 (99.57–99.99)	99.93 (99.60–99.99)	0.9945
N	0	1315					
IBV							
P	6	0	1414	100.00 (60.97–100.00)	100.00 (99.73–100.00)	100.00 (99.73–100.00)	1.0000
N	0	1408					
RSV							
P	76	0	1414	100.00 (95.19–100.00)	100.00 (99.71–100.00)	100.00 (99.73–100.00)	1.0000
N	0	1338					
SARS‐CoV‐2							
P	368	5	1438	99.46 (98.05–99.85)	99.53 (98.91–99.80)	99.51 (99.00–99.76)	0.9873
N	2	1063					

*Note:* Values in parentheses are two‐sided 95% confidence intervals (Wilson score). The reference standard was real‐time fluorescence quantitative PCR, and discordant results were adjudicated by Sanger sequencing.

Abbreviations: IAV, influenza A virus; IBV, influenza B virus; N, negative; NPA, negative percent agreement; P, positive; PPA, positive percent agreement; RSV, respiratory syncytial virus; TPA, total percent agreement.

### 3.2 LoD

The LoD was established at 100 copies/mL for SARS‐CoV‐2 and 200 copies/mL for IAV, IBV, and RSV. A 100% detection rate was achieved for all four pathogens at their respective LoD concentrations (Table 2), successfully validating alignment with the manufacturer's claimed specifications.

**TABLE 2:** Data of LoD.

Pathogens	Concentration (copies/mL)	Number	Detection rate	Range of Ct value	CV of Ct value
SARS‐CoV‐2	100	20/20	100%	36.31–39.08	2.02%
IAV	200	20/20	100%	36.28–38.32	1.56%
IBV	200	20/20	100%	35.31–37.09	1.28%
RSV	200	20/20	100%	36.13–38.76	1.61%

Abbreviations: Ct, cycle threshold; CV, coefficient of variation; IAV, influenza A virus; IBV, influenza B virus; RSV, respiratory syncytial virus.

### 3.3 Repeatability

A 100% detection rate was achieved for both negative and positive nucleic acid samples across all four viruses, with Ct value CVs consistently below 3% (Figure 1 and Table 3), confirming successful verification of assay performance. Three samples per positive concentration level were tested in 10 replicates each; Table 3 presents one representative sample per level, and the complete dataset is shown in Figure 1.

**FIGURE 1:**
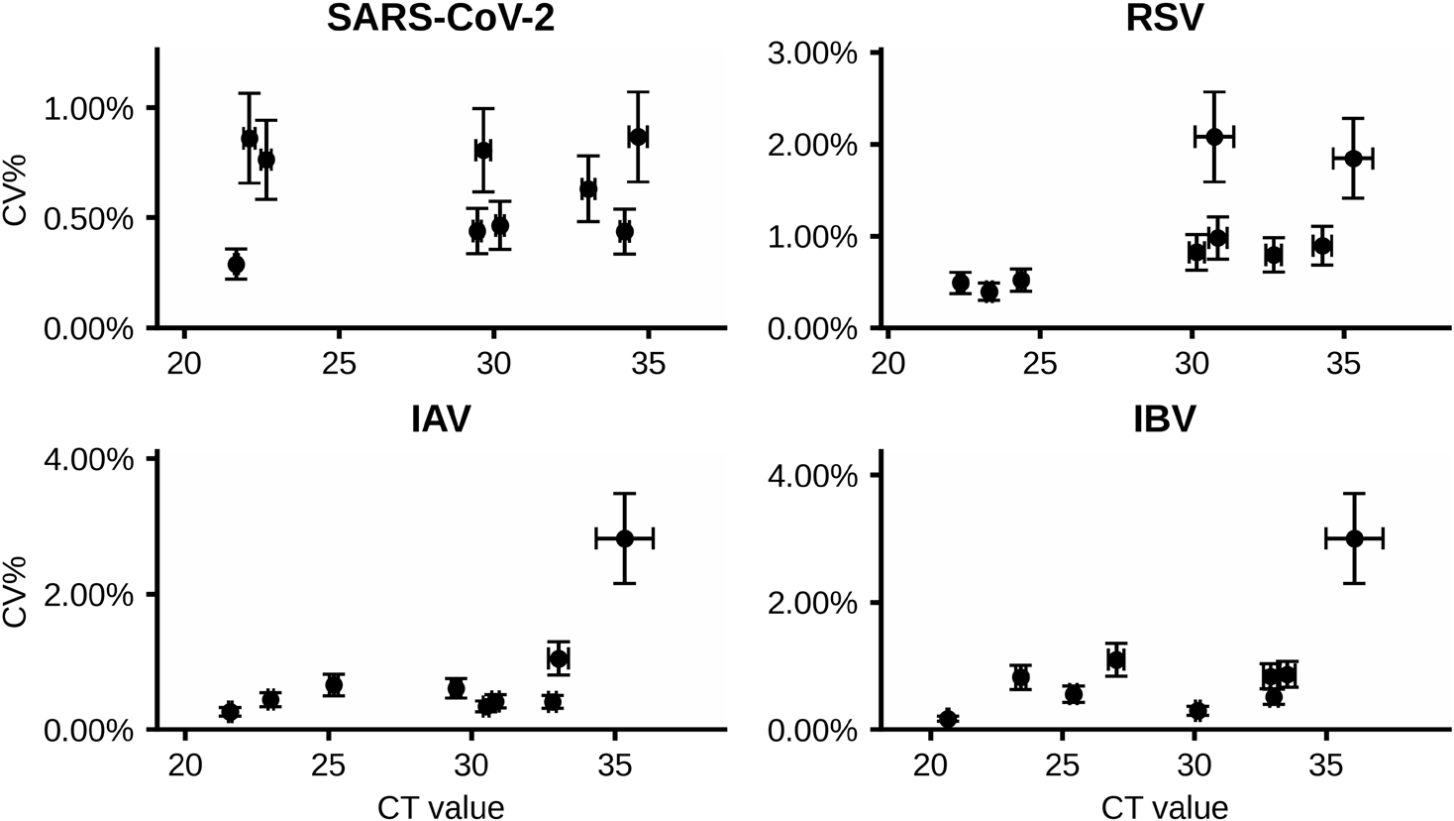
Repeatability. Repeatability was assessed using three independent samples per positive concentration level (low/medium/high viral load) and one negative sample for each of the four targets (SARS‐CoV‐2, RSV, IAV, IBV), with 10 replicates per sample. Intra‐assay (within‐run) repeatability is reported as mean Ct ± SD and mean CV% ± SD. Ct, cycle threshold; CV, coefficient of variation; IAV, influenza A virus; IBV, influenza B virus; RSV, respiratory syncytial virus; SD, standard deviation.

**TABLE 3:** Data of repeatability.

Pathogens	Number	Detection rate	Range of Ct value	Mean of Ct	SD of Ct	CV of Ct value
SARS‐CoV‐2						
H	10/10	100%	21.59–21.77	21.68	0.059	0.27%
M	10/10	100%	29.34–29.72	29.46	0.123	0.12%
L	10/10	100%	33.97–34.39	34.23	0.142	0.41%
N	10/10	100%	—	—	—	—
IAV						
H	10/10	100%	21.48–21.65	21.57	0.053	0.25%
M	10/10	100%	29.06–29.67	29.46	0.169	0.57%
L	10/10	100%	34.41–37.75	35.34	0.945	2.67%
N	10/10	100%	—	—	—	—
IBV						
H	10/10	100%	20.59–20.69	20.66	0.033	0.16%
M	10/10	100%	30.03–30.35	30.13	0.083	0.28%
L	10/10	100%	33.04–34.02	33.53	0.275	0.82%
N	10/10	100%	—	—	—	—
RSV						
H	10/10	100%	22.23–22.54	22.38	0.104	0.46%
M	10/10	100%	29.62–30.41	30.16	0.235	0.78%
L	10/10	100%	33.83–34.89	34.29	0.290	0.85%
N	10/10	100%	—	—	—	—

*Note:* For each target and concentration level, three samples were tested in 10 replicates each; data for one representative sample are shown.

Abbreviations: Ct, cycle threshold; CV, coefficient of variation; H, high concentration; IAV, influenza A virus; IBV, influenza B virus; L, low concentration; M, medium concentration; N, negative; RSV, respiratory syncytial virus; SD, standard deviation.

### 3.4 Precision

Precision verification demonstrated 100% detection rates for both positive (Target) and negative (Internal Control) nucleic acid samples across all four viruses, with CVs for Ct values consistently below 5% (Figure 2 and Table 4), confirming successful verification of assay precision.

**FIGURE 2:**
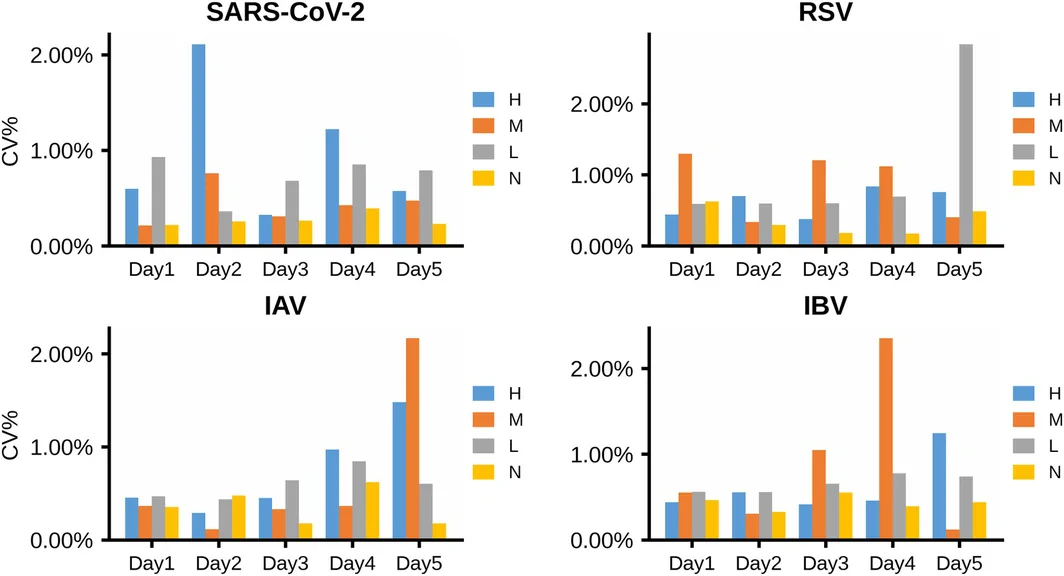
Precision. For each of the four viruses (SARS‐CoV‐2, RSV, influenza A, and influenza B), one sample at each concentration level (negative, low, medium, and high viral load) was tested with five replicates per sample over five consecutive days to assess precision. For negative samples, the internal control (IC) signal is shown. CV, coefficient of variation; H, high; IAV, influenza A virus; IBV, influenza B virus; L, low; M, medium; N, negative; RSV, respiratory syncytial virus.

**TABLE 4:** Data of precision.

Pathogens	Number	Detection rate	Range of Ct value	Mean of Ct	SD of Ct	CV of Ct value
SARS‐CoV‐2						
H	25/25	100%	24.30–25.77	25.38	0.274	1.08%
M	25/25	100%	28.44–29.02	28.64	0.129	0.45%
L	25/25	100%	32.47–33.59	33.05	0.238	0.72%
N	25/25	100%	26.46–26.91	26.72	0.107	0.40%
IAV						
H	25/25	100%	24.56–25.45	24.98	0.200	0.80%
M	25/25	100%	28.49–30.18	29.81	0.299	1.00%
L	25/25	100%	32.54–33.48	33.07	0.198	0.60%
N	25/25	100%	26.78–27.50	27.05	0.166	0.61%
IBV						
H	25/25	100%	23.58–24.40	24.11	0.160	0.67%
M	25/25	100%	28.77–30.70	30.29	0.356	1.18%
L	25/25	100%	32.76–33.63	33.19	0.208	0.63%
N	25/25	100%	26.73–27.43	27.07	0.173	0.64%
RSV						
H	25/25	100%	25.65–26.55	26.03	0.229	0.88%
M	25/25	100%	29.52–30.60	30.32	0.271	0.89%
L	25/25	100%	31.60–34.28	33.76	0.489	1.45%
N	25/25	100%	26.90–26.96	26.77	0.103	0.39%

*Note:* For negative (N) samples, the internal control (IC) Ct values are reported.

Abbreviations: Ct, cycle threshold; CV, coefficient of variation; H, high concentration; IAV, influenza A virus; IBV, influenza B virus; L, low concentration; M, medium concentration; N, negative; RSV, respiratory syncytial virus; SD, standard deviation.

### 3.5 Cross‐Reaction

All cross‐reactivity testing panels returned negative results in triplicate replicates, confirming successful verification of assay specificity (Table 5).

**TABLE 5:** Result of cross‐reaction.

Mixed sample	Ct value (Target)			Detection result
	First	Second	Third	
Rhinovirus	—	—	—	N
Parainfluenza virus	—	—	—	N
Adenovirus	—	—	—	N
*Mycoplasma pneumoniae*	—	—	—	N
*Haemophilus influenzae*	—	—	—	N
*Staphylococcus aureus*	—	—	—	N

Abbreviations: Ct, cycle threshold; N, negative.

### 3.6 Interference Resistance

Weakly‐positive samples spiked with seven distinct interferents demonstrated consistent detection results compared to unspiked controls, with all replicates maintaining weakly‐positive status (Table 6). This confirms successful verification of interference resistance. Across the 28 valid target‐level pairs, the mean ΔCt was 2.38 ± 1.36 (range: −0.95 to 4.55). Although the paired *t*‐test indicated a statistically significant systematic increase (*t* = 9.26, *p* < 0.001), all spiked samples were still correctly detected as low‐positive, indicating that the observed shift does not affect qualitative result interpretation.

**TABLE 6:** Result of interference resistance.

Interferent	SARS‐CoV‐2 (Mean of Ct/Result)		IAV (Mean of Ct/Result)		IBV (Mean of Ct/Result)		RSV (Mean of Ct/Result)	
Human blood								
Control	33.43	WP	33.71	WP	32.85	WP	34.56	WP
Interferent	36.42	WP	35.34	WP	35.86	WP	37.12	WP
Mucin								
Control	33.40	WP	33.39	WP	32.95	WP	35.89	WP
Interferent	36.42	WP	35.34	WP	35.86	WP	37.12	WP
Oseltamivir								
Control	33.22	WP	32.68	WP	32.35	WP	33.22	WP
Interferent	36.78	WP	36.66	WP	36.90	WP	36.51	WP
Peramivir								
Control	33.22	WP	32.68	WP	32.35	WP	34.67	WP
Interferent	35.81	WP	35.99	WP	36.00	WP	35.57	WP
Ribavirin								
Control	33.22	WP	33.03	WP	32.35	WP	35.43	WP
Interferent	33.27	WP	35.51	WP	34.48	WP	34.48	WP
Ceftriaxone								
Control	33.57	WP	33.03	WP	32.74	WP	35.43	WP
Interferent	36.65	WP	36.92	WP	37.06	WP	36.54	WP
Levofloxacin								
Control	33.57	WP	33.57	WP	32.74	WP	35.43	WP
Interferent	34.57	WP	35.64	WP	35.01	WP	35.49	WP

*Note:* ΔCt, mean Ct of the interferent‐spiked sample minus that of the paired control; across 28 valid target‐level pairs, the mean ΔCt was 2.38 ± 1.36 (paired *t*‐test, *t* = 9.26, *p* < 0.001).

Abbreviations: Ct, cycle threshold; IAV, influenza A virus; IBV, influenza B virus; RSV, respiratory syncytial virus; WP, weakly positive.

### 3.7 Daily Quality Control

A total of 115 consecutive experimental runs were verified, with each run incorporating one negative control and one positive control, demonstrating 100% expected results: all 115 negative controls returned no detectable signals, while all 115 positive controls exhibited accurate amplification, verifying full compliance with daily QC protocols. As shown in Figure 3, the Ct value variation curves for four positive quality control materials (IAV, IBV, RSV, and SARS‐CoV‐2) demonstrated average Ct values of 32.82 ± 0.95, 32.22 ± 0.79, 32.13 ± 0.66, and 30.41 ± 0.45, respectively. The corresponding CV values were 2.90%, 2.44%, 2.06%, and 1.47%, respectively. The 115 runs covered the entire study period (May 23 to November 19, 2025), and all runs are plotted in the Levey‐Jennings charts in Figure 3. Because the positive control is a medium‐concentration material with a release range of 27 < Ct ≤ 33, Ct values slightly above 33 were observed in some runs (IAV: 39/115; IBV: 29/115; RSV: 16/115; SARS‐CoV‐2: 0/115). Under the applied Westgard rules, these runs triggered only 1_2s warnings (3, 1, 3, and 0 runs for IAV, IBV, RSV, and SARS‐CoV‐2, respectively), and no 1_3s, 2_2s, or R_4s rejection rule was violated. A downward step shift of approximately 1 Ct observed after run 37 was associated with replacement of the quality‐control material lot; control limits were calculated per lot, and all subsequent results remained in control.

**FIGURE 3:**
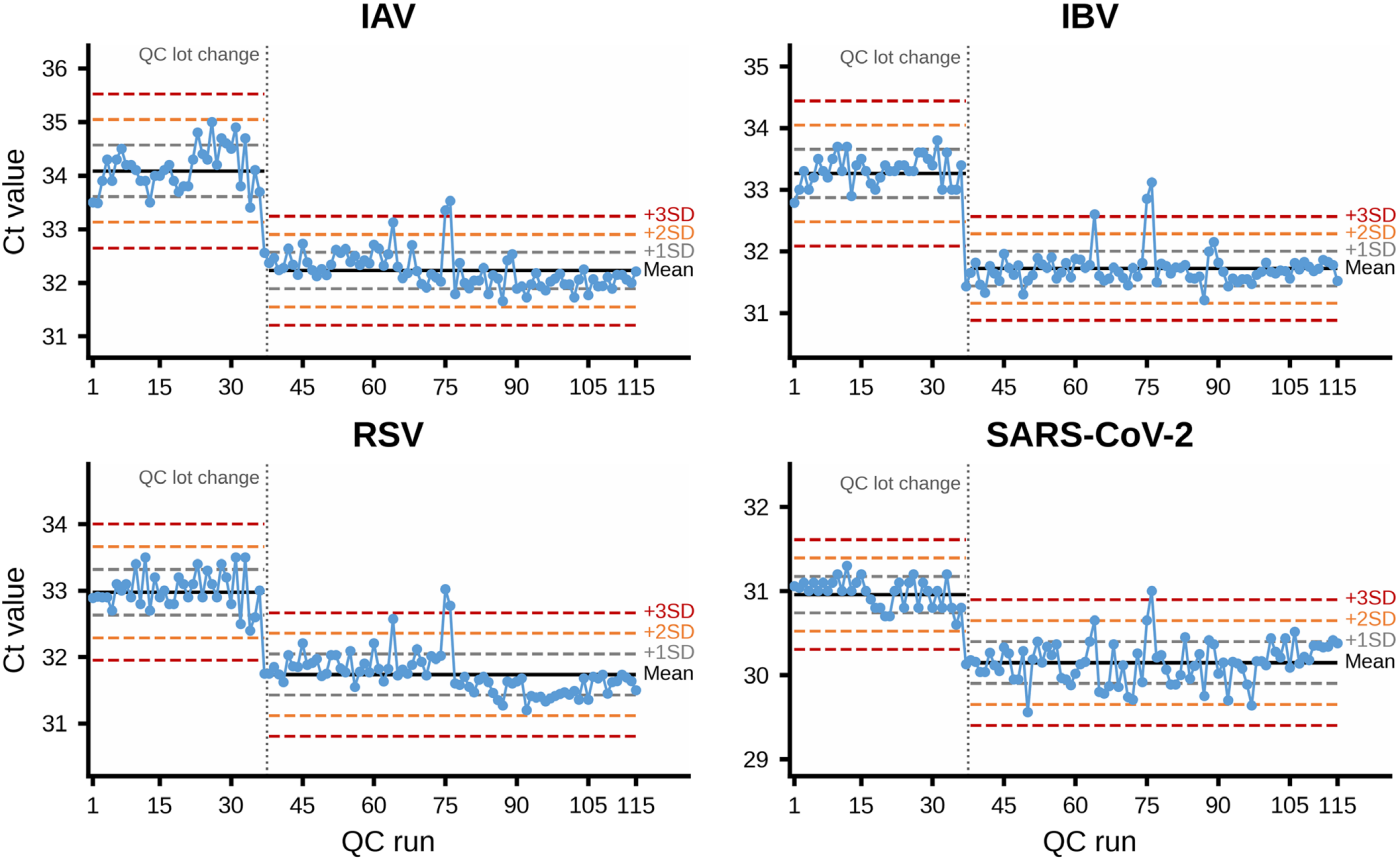
Daily quality control. Levey‐Jennings charts of positive‐control Ct values over 115 consecutive runs (May 23 to November 19, 2025) for the four viruses (SARS‐CoV‐2, RSV, IAV, and IBV). Solid line, mean; dashed lines, ±1SD, ±2SD, and ±3SD control limits calculated per quality‐control lot. Westgard multirules (1_2s warning; 1_3s, 2_2s, and R_4s rejection) were applied. Ct, cycle threshold; IAV, influenza A virus; IBV, influenza B virus; RSV, respiratory syncytial virus.

### 3.8 Contamination Prevention

Respiratory virus nucleic acids analysis was performed on 72 samples obtained from equipment interiors via environmental settling plates. No target amplification was detected in any test sample (72/72 negatives), confirming 100% contamination‐free performance.

## 4 Discussion

### 4.1 Principal Findings

This study verified the performance of the Mindray Fully Automated Molecular System MN2880 for detecting four common respiratory viruses, demonstrating full compliance with CNAS‐GL037:2019 and CNAS‐GL039:2019 through verified parameters: concordance ≥ 99% versus real‐time fluorescence quantitative PCR, LoD ≤ 200 copies/mL, repeatability (100% detection rate for both negative and positive nucleic acid samples), precision (CV ≤ 5%), cross‐reactivity (zero interference from 6 pathogens), interference resistance (verified against 7 interferents), daily QC (100% validity across 115 runs), and carryover prevention (0% contamination in 72 tests), confirming the system is qualified for clinical diagnostics.

### 4.2 Comparison With Conventional PCR and Published POCT Platforms

In the field of acute respiratory infection pathogen diagnosis, high‐throughput conventional PCR systems and rapid POCT platforms represent two distinct technological approaches, exhibiting significant differences in throughput, processing speed, automation level, operational costs, spatial requirements, and operator skill demands. Traditional large‐scale PCR instruments, such as the ABI 7500 or Bio‐Rad CFX96, are typically deployed in centralized laboratories, where their core strengths lie in high‐throughput processing capacity and highly automated sample handling workflows, enabling large‐scale batch testing of numerous specimens [23]. However, such systems typically require complex specimen preprocessing steps and exhibit extended testing cycles, with the turnaround time (TAT) from sample receipt to result reporting often spanning several hours or longer [24]. In contrast, POCT systems exemplified by the Mindray MN2880 demonstrate rapid detection speed as a defining advantage, with most platforms completing testing within 30–60 min, significantly shortening TAT and enabling timely clinical decision‐making [25].

In core performance metrics, specifically sensitivity and specificity, a trade‐off relationship typically exists between platforms. Conventional laboratory PCR, optimized and validated over time, is often regarded as the gold standard with exceptionally high sensitivity and specificity, reliably detecting low‐copy pathogen nucleic acids [26]. To achieve rapid testing speeds, simplified workflows, and compact designs, POCT systems may accept marginal compromises in absolute sensitivity. For instance, certain real‐time fluorescent PCR‐based POCT systems demonstrate LoDs around 1000 copies/mL for most respiratory pathogens, whereas laboratory PCR may achieve lower detection limits [27]. However, such compromises must still meet clinical diagnostic minimum requirements. The Mindray MN2880 system achieves an LoD of 100 copies/mL. Extensive studies confirm that well‐designed POCT molecular platforms exhibit high concordance with laboratory PCR in clinical validation (28; 29), one evaluation showed 90.0% positive agreement, 100% negative agreement, and 97.9% overall concordance versus conventional RT‐PCR [28]. A prospective validation of the SARS‐CoV‐2 POCT reported 100% clinical sensitivity, 99.4% specificity, and 99.4% accuracy [29]. These data indicate that while POCT absolute LoD may slightly trail top‐tier laboratory equipment, its clinical sensitivity remains sufficient to effectively identify infectious patients (typically associated with higher viral loads) [30]. Consequently, platform selection requires balancing clinical scenarios: during outbreaks, emergency triage, or when rapid infection control is critical, POCT systems enable early diagnosis and timely intervention through speed and accessibility, with performance fully supporting clinical decisions [27, 31]. Conversely, high‐throughput conventional PCR excels in large‐scale epidemiological screening, asymptomatic surveillance, or research contexts demanding ultralow LoD detection. A structured comparison of the key performance indicators of the MN2880 system with those of previously reported molecular POCT platforms is provided in Table 7.

**TABLE 7:** Comparison of key performance indicators between the MN2880 system and previously reported molecular POCT platforms for respiratory virus detection.

Platform	Detected targets	Turnaround time	LoD (copies/mL)	Reported agreement (References)
Mindray MN2880	SARS‐CoV‐2, IAV, IBV, RSV	~40 min	100 (SARS‐CoV‐2); 200 (IAV/IBV/RSV)	PPA 99.46%–100%, NPA 99.53%–100% vs. reference real‐time PCR
Xpert Xpress SARS‐CoV‐2/Flu/RSV	SARS‐CoV‐2, IAV, IBV, RSV	~30 min	NR	High concordance with laboratory PCR [8, 10]
STANDARD M10 Flu/RSV/SARS‐CoV‐2 Fast	SARS‐CoV‐2, IAV, IBV, RSV	~36 min	NR	Sensitivity 98.2%–100%, high specificity [14, 15]
PowerChek Respiratory Virus Detection Assay	Multiple respiratory viruses	NR	NR	Accuracy > 94.6%; kappa 0.843–1.000 vs. BioFire RP 2.1plus/Allplex RP [32]
BD MAX	SARS‐CoV‐2	NR	NR	100% concordance vs. laboratory‐developed RT‐PCR [33]

Abbreviations: IAV, influenza A virus; IBV, influenza B virus; LoD, limit of detection; NPA, negative percent agreement; NR, not reported in the cited reference; PPA, positive percent agreement; RSV, respiratory syncytial virus.

### 4.3 Discordant Results and Their Management

Despite achieving satisfactory concordance, the Mindray MN2880 assay yielded two false‐negative and five false‐positive SARS‐CoV‐2 detections. These accuracy deviations reflect inherent systemic vulnerabilities in the end‐to‐end testing process. False negative, attributable to low viral loads, compromised sample integrity, PCR inhibitors, or viral genetic variations [34, 35], poses critical risks through undiagnosed infections and subsequent transmission. False positive, predominantly induced by amplicon contamination or off‐target amplification [36, 37], may lead to unnecessary interventions and resource strain. Consequently, rigorous pre‐analytical sample management constitutes the cornerstone for ensuring the accuracy of SARS‐CoV‐2 detection results on the Mindray MN2880 nucleic acid testing platform. Furthermore, establishing and strictly implementing standardized operating procedures (SOPs) for sample collection, preservation, and transportation is critical. A robust laboratory quality control system serves as an essential safeguard against false positives by preventing cross‐contamination. Ultimately, optimizing testing workflows and implementing judicious interpretive criteria represent the ultimate measures to address equivocal results and minimize diagnostic errors. In this study, all discordant specimens (seven for SARS‐CoV‐2 and one for IAV) were retested in duplicate on both platforms and adjudicated by Sanger sequencing, and the adjudicated results were used for the final classification shown in Table 1.

### 4.4 Performance in the Context of Previous Studies

Clinical sample concordance verification constitutes a critical performance assessment for ARI virus nucleic acid detection systems, centered on blinded testing of clinical remnant specimens or standardized panels with known pathogen status to calculate positive percent agreement (PPA), negative percent agreement (NPA), and overall concordance. A previous study evaluating the PowerChek Respiratory Virus Detection Assay, analysis of 336 nasopharyngeal swabs demonstrated > 94.6% accuracy versus the reference method BioFire RP 2.1plus and Allplex RP assays, with Cohen's kappa values ranging from 0.843 to 1.000, confirming robust PPA/NPA [32]. A separate SARS‐CoV‐2 evaluation comparing the BD MAX system against laboratory‐developed real‐time RT‐PCR across 300 throat swabs and 100 sputum samples established 100% concordance, validating high clinical agreement in this automated platform [33]. For the Mindray MN2880 detection system, PPA, NPA, and TPA exceed 99%, with Cohen's kappa coefficient demonstrating excellent agreement (> 0.98).

LoD remains a pivotal performance metric for viral nucleic acid detection systems. A previous study developing a multiplex PCR method with fluorescence melting curve analysis for simultaneous detection of six respiratory pathogens demonstrated high sensitivity, with LoDs ranging from 4.94 to 14.03 copies/μL across targets during analytical validation [38]. Similarly, a multiplex one‐step RT‐PCR‐lateral flow assay for seven respiratory pathogens reported the LoD of 10 copies/μL [39]. Notably, LoD varies significantly across technological platforms. A spatially encoded microfluidic chip integrating CRISPR‐Cas12a and recombinase polymerase amplification achieved ultra‐low LoDs of approximately 1 copy/reaction for nine respiratory viruses [40], while a quantum dot nanobead‐based multiplex fluorescent lateral flow immunosensor elevated sensitivity by 100 to 1000‐fold over traditional colloidal gold strips, with LoDs of 56, 120, and 41 copies/mL for SARS‐CoV‐2, adenovirus, and IAV, respectively [41]. These findings underscore that rigorous LoD validation, using platform‐appropriate reference materials and statistical methods, is essential when evaluating systems like the MN2880, ensuring reliable detection in clinical low‐viral‐load specimens and preventing false negatives. Our results confirm the MN2880 system's LoD of 100–200 copies/mL, aligning closely with the aforementioned reports [38, 41].

Repeatability (within‐run precision) constitutes a critical performance metric for ARI virus nucleic acid detection systems, directly reflecting result stability and consistency under strictly controlled identical conditions. For qualitative systems detecting SARS‐CoV‐2, influenza, or RSV, repeatability validation requires 100% positive detection rates across repeated tests, ensuring reliable identification of positive samples near the LoD and preventing false negative [42]. For quantitative/semi‐quantitative systems, validation necessitates calculating the mean, standard deviation, and CV of replicate Ct measurements. The CV, a key precision parameter, must meet manufacturer claims or industry standards, typically requiring < 5% at moderate concentrations [43]. A study of the Savanna RVP4 system, its repeatability assessment involved triplicate same‐day testing of SARS‐CoV‐2, IAV, and RSV samples, yielding within‐run CVs of 3%, 5%, and 0%, respectively [44]. These low CV values demonstrate exceptional analytical precision and system stability. Our study confirmed that the MN2880 system achieves 100% detection rates for both positive and negative samples across four target respiratory viruses in repeatability and precision testing, with CV of Ct consistently below 5%.

Interference resistance validation is a critical component in evaluating ARI virus nucleic acid detection systems, focusing on assessing the impact of common endogenous and exogenous substances on test results. Endogenous interferents originate from sample matrices, such as blood, mucin, and nasal secretion components, which may compromise accuracy by inhibiting polymerase activity, impeding nucleic acid extraction, or generating nonspecific signals [45]. Exogenous interference involves substances introduced during sampling/handling, including common medications and additives in transport media [46]. A study on SARS‐CoV‐2‐rapid nucleic acid detection systems demonstrated robustness by confirming unaffected positive detection in samples spiked with potential interferents [45]. While closed automated systems physically minimize operator‐induced contamination, their biochemical reaction systems still require rigorous validation against matrix‐derived interference [47]. The MN2880 system successfully passed interference resistance validation, yielding identical low‐positive results for samples containing seven distinct interferents compared to uncontaminated controls.

### 4.5 System Design, Strengths, and Limitations

The MN2880 system's highly integrated design minimizes error‐prone manual handling inherent in traditional molecular testing, enhancing process standardization and reproducibility. Its fully automated workflow, from sample loading to result reporting, increases throughput while reducing operational variability. Testing occurs within enclosed modular units, effectively mitigating result fluctuations caused by human intervention. This closed‐system architecture theoretically reduces amplicon contamination risks, a critical advantage for high‐sensitivity respiratory virus nucleic acid detection where false positives must be prevented. By avoiding nonspecific amplification from environmental or cross‐contamination, the system ensures reliable results. These attributes enable the MN2880 to meet stringent biosafety and accuracy requirements during urgent, large‐scale screenings for infectious pathogens like SARS‐CoV‐2.

Several limitations of this study should be acknowledged. First, this was a single‐center verification performed with one instrument and a single reagent lot, so between‐lot and between‐instrument variability could not be assessed. Second, only oropharyngeal swabs were evaluated, and performance with other specimen types (e.g., nasopharyngeal swabs) requires further study. Third, the number of IBV‐positive specimens was small (*n* = 6) owing to the low prevalence of influenza B during the study period; consequently, the lower 95% confidence limit of the IBV positive percent agreement (60.97%) did not meet the prespecified > 90% criterion, and IBV positive agreement should be confirmed in larger cohorts. Fourth, although the cross‐reactivity panel covered six clinically relevant pathogens at high concentrations, it could not include all potentially cross‐reacting organisms; in silico analysis was therefore used to complement the wet‐laboratory panel. Fifth, carryover was assessed indirectly through environmental settle‐plate sampling rather than a dedicated alternating high‐titer carryover experiment. Finally, statistically significant Ct shifts were observed for several interferents; although qualitative results were unaffected at the concentrations tested, caution is warranted when interpreting specimens with viral loads close to the LoD.

## 5 Conclusions

The Mindray MN2880 integrated system demonstrates compliance with all critical performance metrics for detecting four major respiratory viral nucleic acids, establishing its suitability for routine clinical diagnostics. Beyond these verified capabilities, the platform offers four key advantages: First, the entire testing process from sample‐to‐result occurs within a single device, eliminating traditional molecular lab partitioning requirements. Second, all reactions are contained within a sealed module without manual lid removal, minimizing operator biohazard exposure. Third, total processing time of approximately 40 min outperforms conventional real‐time PCR, enabling POCT nucleic acid testing ideal for fever clinics, emergency treatment, and intensive care units. Fourth, occupying < 1 m^2^, the system allows immediate deployment in primary hospitals lacking molecular lab infrastructure. The MN2880 operational simplicity, rapid reporting, and minimal spatial demands significantly enhance nucleic acid testing accessibility and clinical responsiveness, proving critical for rapid pathogen identification in respiratory infections.

## Author Contributions

J.C., H.H., and P.C. conceived, designed the study, and wrote. J.C. and L.Z. performed all the experiments. J.C., L.Z., and H.H. prepared all the figures and analyzed the data. J.C., L.Z., H.H., and P.C. investigated and reviewed the manuscript. H.H. and P.C. supervised the study. All authors agree to be accountable for all aspects of work ensuring integrity and accuracy.

## Funding

The authors have nothing to report.

## Ethics Statement

The study was approved by the Ethics Committee of the First Affiliated Hospital of Sun Yat‐sen University (Ethics Approval: No. 2024‐554).

## Consent

The requirement for written informed consent was waived by the committee because only deidentified residual specimens from routine clinical testing were used.

## Conflicts of Interest

Yuping He is an employee of Shenzhen Mindray Bio‐Medical Electronics Co. Ltd., which provided technical and resource support for this study. The other authors declare no conflicts of interest.

## Data Availability

The data that support the findings of this study are available from the corresponding author upon reasonable request.
